# Nanoparticle Emulsions Enhance the Inhibition of NLRP3

**DOI:** 10.3390/ijms231710168

**Published:** 2022-09-05

**Authors:** Minjie Cao, Luyun Cai

**Affiliations:** 1 Ningbo Research Institute, College of Biosystems Engineering and Food Science, Zhejiang University, Ningbo 315100, China; 2 College of Biological and Chemical Engineering, Zhejiang Engineering Research Center for Intelligent Marine Ranch Equipment, NingboTech University, Ningbo 315000, China

**Keywords:** antimicrobial peptide, Pickering emulsion, peritonitis, NLRP3

## Abstract

Antibacterial delivery emulsions are potential materials for treating bacterial infections. Few studies have focused on the role and mechanism of emulsions in inflammation relief. Therefore, based on our previous analysis, in which the novel and natural Pickering emulsions stabilized by antimicrobial peptide nanoparticles were prepared, the regulation effect of emulsion on inflammasome was explored in silico, in vitro and in vivo. Firstly, the interactions between inflammasome components and parasin I or Pickering emulsion were predicted by molecular docking. Then, the inflammasome stimulation by different doses of the emulsion was tested in RAW 264.7 and THP-1 cells. Finally, in Kunming mice with peritonitis, NLRP3 and IL-1β expression in the peritoneum were evaluated. The results showed that the Pickering emulsion could combine with ALK, casp-1, NEK7, or NLRP3 to affect the assembly of the NLRP3 and further relieve inflammation. LPNE showed a dose–dependent inhibition effect on the release of IL-1β and casp-1. With the concentration of parasin I increased from 1.5 mg/mL to 3 mg/mL, the LDH activity decreased in the chitosan peptide-embedded nanoparticles emulsion (CPENE) and lipid/peptide nanoparticles emulsion (LPNE) groups. However, from 1.5 to 6 mg/mL, LPNE had a dose–dependent effect on the release of casp-1. The CPENE and parasin I-conjugated chitosan nanoparticles emulsion (PCNE) may decrease the release of potassium and chloride ions. Therefore, it can be concluded that the LPNE may inhibit the activation of the inflammasome by decreasing LDH activity, potassium and chloride ions through binding with compositions of NLRP3.

## 1. Introduction

The fatality rate of peritonitis caused by severe *E. coil* infection is high. Infection can cause further sepsis and multiple organ failure with a fatality rate of more than 30%. Therefore, antibacterial drugs play a pivotal role in treating abdominal infections. However, due to antibiotic resistance, the rapidly emerging global health problems have brought significant challenges to microbial infection [1,2,3]. It was recently estimated that millions of people worldwide might die from sepsis every year due to antibiotic-resistant infections [4].

There are multiple strategies to overcome problems with resistance of microorganisms, such as decreasing the usage of antibiotics, using antimicrobial peptides, exploring novel antimicrobial reagents, etc. Antimicrobial peptides have aroused widespread concern due to their complex development of drug resistance, and solid antibacterial and immunomodulatory ability [5,6,7]. Some promising antimicrobial peptides can replace classic antibiotics for drug-resistant infections, such as pexiganan acetate, Omiganan, and Dulaglutide. They exhibit high activity against Gram-positive and Gram-negative bacteria, and they can target bacterial, fungal, parasitic, and eukaryotic cells indiscriminately [8,9]. However, limited success in the clinical application of these peptides urgently needs to be resolved, such as high hemolysis toward human cells, short circulating plasma half-life, easily changing the homeostasis of intestinal microflora, poor in vivo stability, and so on [10]. In our previous study, three kinds of oil in water Pickering emulsions stabilized with solid particles (chitosan peptide-embedded nanoparticles Pickering emulsion (CPENE), parasin I-conjugated chitosan nanoparticles Pickering emulsion (PCNE), lipid/peptide nanoparticles Pickering emulsion (LPNE)) successfully improved the poor stability, high hemolysis, and high toxicity in a mouse model of peritonitis. However, how Pickering emulsions decrease the symptoms of inflammation are unclear. More in-depth explorations of anti-inflammatory mechanisms are needed.

The aberrant activation of NLR family, pyrin domain-containing 3 (NLRP3) inflammasomes—a protein complex assembled of NLRP3, apoptosis-associated speck-like protein (ASC), and cysteinyl aspartate specific proteinase (casp-1)—contributes to the development of peritonitis. The NLRP3 inflammasome can activate casp-1, produce functional interleukin-1β (iL-1β), and further induce cell apoptosis. It is reported that many compounds have high anti-inflammatory activity and are beneficial to NLRP3-related diseases [11,12,13,14,15,16,17,18]. However, many antimicrobial peptides from food show low anti-inflammatory activity and destroy the colonization of beneficial bacteria [19]. The Pickering emulsion may change the interactions between inflammasome components and peptides through the interfacial effect of emulsion and the interference effect of biomacromolecules. However, little research addressed the function of the Pickering emulsion in inflammasome inhibition. The Kunming mice have been widely used as a bacterial peritonitis mouse model in research due to their advantages: good reproductive performance, fast growth, strong disease resistance and biological characteristics similar to humans and other mammals in the natural state [20,21].

Therefore, this study predicted the interactions between inflammasome components and parasin I or Pickering emulsion. Furthermore, the mechanism of Pickering emulsion inhibition on the activation of the inflammasome was explored in inflammation cells and Kunming mice. This research may provide knowledge about the function of Pickering emulsions on NLRP3 inhibition in inflammation-related diseases.

## 2. Results and Discussions

The material characterization, including its composition, size, surface properties, degradation properties, etc., was provided in our previous publication [22]. In LPN, the parasin I is embedded in the lecithin. In CPEN, parasin I was encapsulated by thiolated chitosan. In PCN, the parasin I was conjugated with chitosan through a C−N bond. Three nanoparticles are formed by self-assembly or ion cross-linking. Three kinds of emulsion were prepared with a fish oil/aqueous phase ratio of 92/8 (*w*/*w*) stabilized by LPN, PCN and CPEN, respectively. The size distribution of emulsion droplets in CPENE, LPNE and PCNE were 90–0.98, 1.03–1.08 and 1.0–1.25 μm, respectively. The composition of Pickering emulsion: parasin-I, chitosan, lecithin and fish oil show higher mucoadhesive properties and can be derived from food. Therefore, these three emulsions are natural food-grade Pickering emulsions.

### 2.1. Molecular Docking

**Scheme 1:**
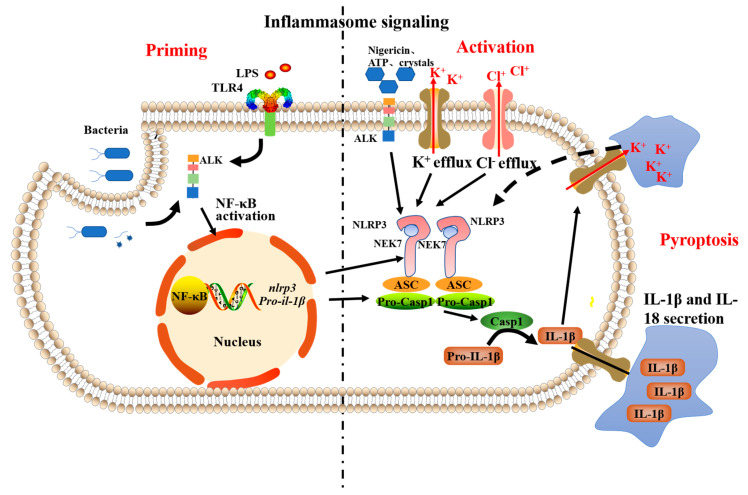
The Priming and Activation of the NLRP3 Inflammasome.

The NLRP3 inflammasome is a complex including NLRP3, ASC, and casp-1, which plays an essential role in the innate immune defense system and can cause cell apoptosis and tissue damage. Many compounds were found essential to the assembly of the inflammasome [23]. As shown in Scheme 1, the ALK is required for NLRP3 inflammasome activation in macrophages [24]. The NEK7 is an essential mediator of NLRP3 activation downstream of potassium efflux [25]. The macrophages stimulated by LPS and ATP can trigger the assembly of NEK7 and NLRP3. Casp-1 is a crucial indicator for detecting cell pyroptosis [26]. The activated inflammasome can modify the casp-1 and the growth of cytokines, and the ripening and secretion of the cells are promoted in the process of natural immune defense. Therefore, tissue damage will be relieved in disease if these signals are blocked.

In this analysis, three kinds of Pickering emulsions (CPENE, LPNE, and PCNE) were prepared to inhibit the activation of the inflammasome and decrease the inflammatory damage of tissue in the peritonitis mouse model. Firstly, to judge if the three kinds of Pickering emulsion can inhibit the activation of the inflammasome, the peptide-conjugated chitosan complex, parasin I, chitosan, thiolated chitosan, and lecithin underwent in silico molecular docking with four inflammasome components: ALK, NEK7, casp-1, and NLRP3.

**Figure 1:**
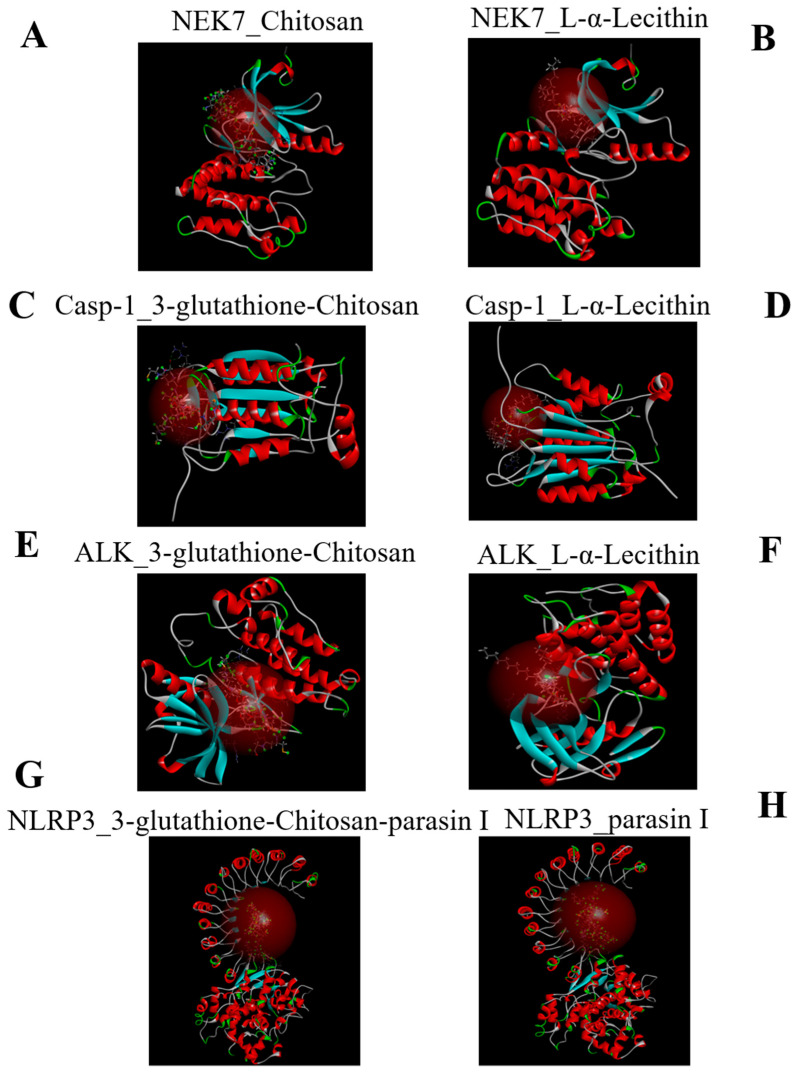
Stereoview of the minimum energy docked conformation of NEK7 with chitosan. (**A**) NEK7 with L-α-lecithin (**B**) casp-1 with 3-glutathione with chitosan (**C**) casp-1 with L-α-lecithin (**D**) ALK with 3-glutathione-chitosan (**E**) ALK with L-α-lecithin (**F**) 6NPY with 3-glutathione-chitosan-parasin I (**G**) 6NPY with parasin I (**H**) during the molecular docking.

Computer simulation aimed to exclude the improper inflammasome inhibitors and discover potential effective inhibitors. Our previous publications verified that parasin I conjugated to chitosan through an amido bond. The ratio of chitosan and parasin I in parasin I-conjugated chitosan matrices was 1:1 [22]. The 3D diagrams of successful docking results are shown in Figure 1. Several donor atoms of NEK7 surrounded the chitosan and lecithin. 3-glutathione -chitosan and L-α-lecithin showed stronger binding with one end of casp-1 and ALK.

Furthermore, the thiolated-chitosan and parasin I can both dock with NLRP3. The lowest binding affinities and binding force types of all dockings are shown in Table 1. There were no hydrogen bonds in any binding. The interactions between NLRP3 and 3-glutathione-chitosan-parasin I (−167 kcal/mol) were predicted to be stronger than that of NLRP3 and parasin I (−138 kcal/mol).

**Figure 2:**
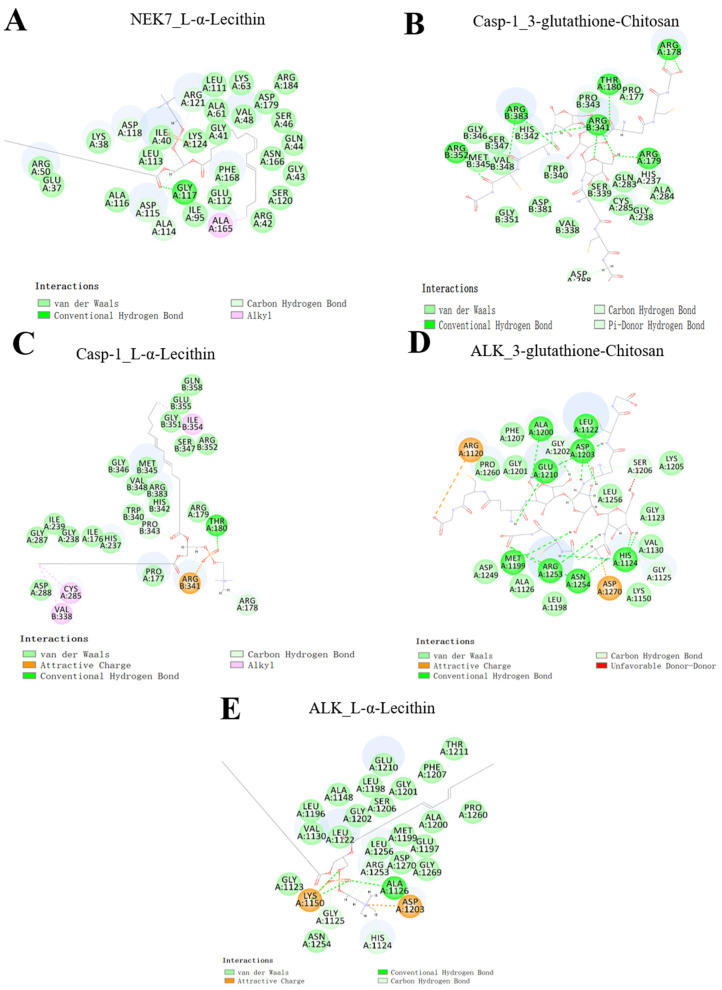
The interaction domain and atoms of the minimum energy docked conformation of components of NLRP3 inflammasome and nanoparticles.

**Scheme 2:**
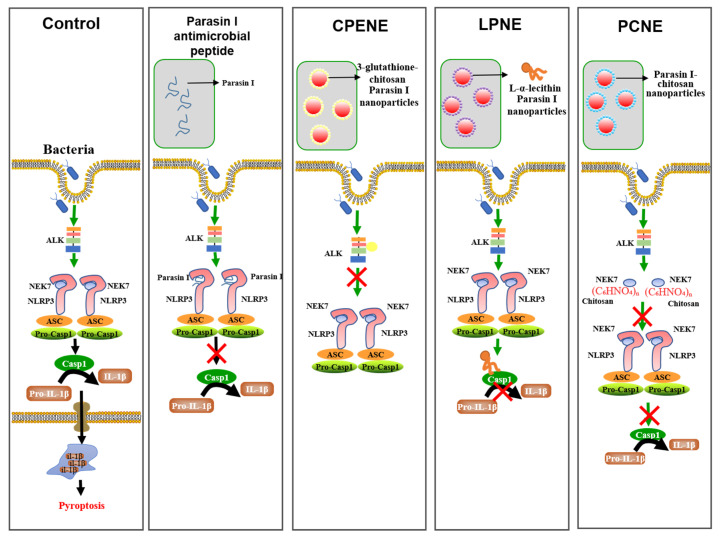
The hypothesis of the effect of Pickering emulsions on the NLRP3 inflammasome activation and IL-1β secretion.

The lipid/peptide adduct will increase the interaction between NLRP3 and parasin I. Compared with L-α-lecithin, which had a CDOCKER_INTERATION_ENERGY of −79 and −70 with NEK7 and casp-1, respectively, the chitosan and chitosan-glutathione-3 had a higher CDOCKER_INTERACTION_ENERGY, which indicated peptide-embedded chitosan matrices and peptide-conjugated chitosan matrices had a more robust interaction with components of inflammation. Although the nanoparticle and Pickering emulsion molecular docking were not directly carried out with inflammasome components, the soft materials and convergent configurations of nanoparticles and Pickering emulsion carrier will enhance the interaction between inflammasome components and 3-glutathione-chitosan-parasin I, chitosan, or lecithin. Yuan et al. [27], also reported that the reconfigurable assembly of colloidal material might cause more adaptation and interactive functions. These bindings with ALK, NEK7, casp-1, and NLRP3 will hinder the combination of the NLRP3 inflammasome. The interaction domain and atoms of the components of NLRP3 inflammasome and nanoparticles are shown in Figure 2 (the ligand in which atoms exceed the maximum number of atoms specified in the preferences is not shown). These results showed that NLRP3 components could interact with GLY, ALA, ARG, ASP, GLU, LEU, HIS and form alkyl, carbon-hydrogen bond, attractive charge, van der Waals, conventional hydrogen bond, pi-donor hydrogen bond with components of the nanoparticles. Therefore, the following hypothesis can be made; parasin I can dock with NLRP3, competing with NEK7 for its locus (Scheme 2). It can further decrease the activity of pro-caspase 1. CPENE affected the NF-κb activation, and the LPNE affected the function of casp-1 and decreased the secretion of IL-1β. PCNE can dock with NEK7, which hinders the binding of NEK7 and NLRP3. All in all, the stronger binding in Pickering emulsion and compositions of NLRP3 can affect the assembly of the NLRP3 and further relieve inflammation.

### 2.2. The NLRP3-Inflammasome Inhibition in Macrophages

**Figure 3:**
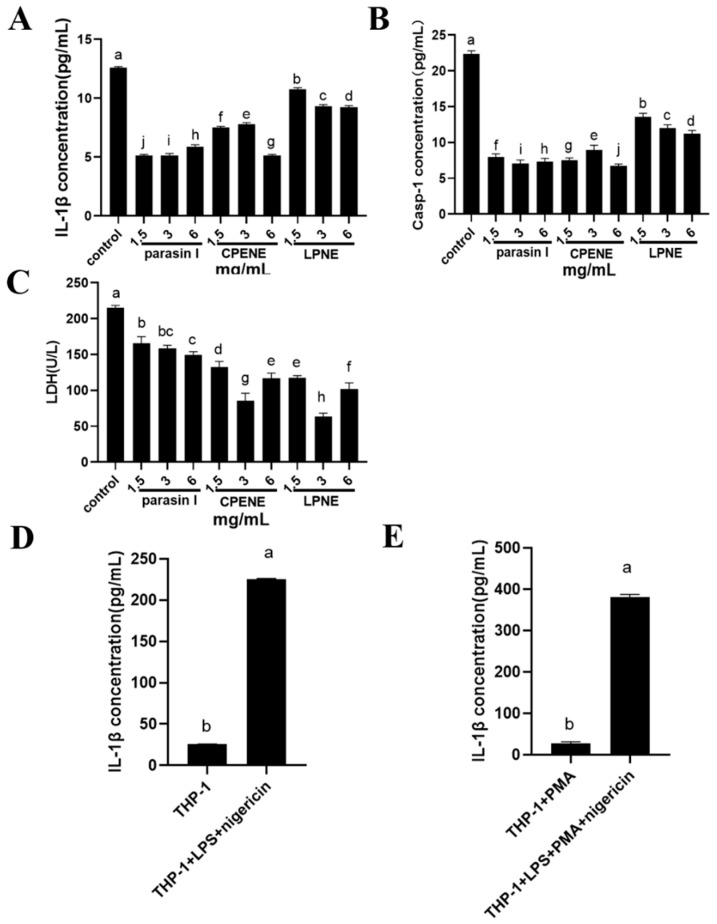
The Pickering emulsion enhances the inhibition effect of Parasin I on activated NLRP3 inflammasome in RAW 264.7. The IL-1β (**A**), casp-1 (**B**) and LDH (**C**) concentration in RAW 264.7, and the induction of THP-1 with PMA (**D**) or without PMA (**E**). different letters (“a”–”j”) indicate significant differences (*p* < 0.05).

Molecular docking results were used as an early screening tool in many studies [28,29,30]. After the prediction of the interactions, to further verify that the Pickering emulsion can block NLRP3 activation, the NLRP3-inflammasome inhibition in macrophages was tested. The mechanism of Pickering emulsion on casp-1 activation and IL-1β release were also explored. It is generally recognized that the activation of the NLRP3 inflammasome needs pre-stimulation and activation processes [31]. Activation of the NF-κB signal pathway and up-regulated expression of NLRP3 and pro-IL-1β were included in the pre-stimulation process (Scheme 1) [24]. Three concentrations (1.5 mg/mL, 3 mg/mL, 6 mg/mL parasin I) of parasin I solution, CPENE, and LPNE Pickering emulsions were incubated with RAW264.7 cells. As shown in Figure 3A,B, when the concentration of parasin I in LPNE increased from 1.5 to 6 mg/mL, the concentration of IL-1β and NLRP3 increased significantly, which indicated the LPNE had a noticeable inhibition effect on IL-1β and NLRP3 secretion. Therefore, LPNE showed a dose–dependent inhibition effect on the release of IL-1β and casp-1, indicating that LPNE might hinder the maturation of IL-1β and casp-1. Compared with the control group, the parasin I solution and CPENE showed lower concentrations of IL-1β and casp-1, which indicated they could inhibit casp-1 activation and IL-1β release stimulated by LPS and nigericin. Parasin I showed a higher inhibition effect than CPENE on IL-1β, which illustrated that the Pickering emulsion did not increase IL-1β release in RAW 264.7 cells. These results may be because the L-α-lecithin can dock with NEK7, casp-1 and ALK, and the 3-glutathione-chitosan can dock with casp-1, ALK and NLRP3. The structure and the interactions with mediators of inflammation were crucial for pro-inflammation [32]. Therefore, the more interactions between the Pickering emulsion and key inflammation compounds, the higher the anti-inflammatory effect of the Pickering emulsion. The Pickering emulsion increased IL-1β release compared with parasin I. This is mainly because the antimicrobial peptide precipitate in the unstable emulsion promotes the cells’ inflammatory response.

Besides the IL-1β and casp-1 release, the cell damage also needs to be analyzed. LDH is an enzyme in the plasma of living cells. When the cell is damaged and the permeability of the cell membrane changes, it will release LDH into the culture medium [33]. Therefore, the enzyme activity in the medium is proportional to the number of lysed cells. In this analysis, the activity was tested to evaluate the cell membrane damage in different groups. As shown in Figure 3C, the LDH activity of Parasin I was higher than CPENE and LPNE. With the concentration of the parasin I increased from 1.5 mg/mL to 6 mg/mL, the LDH activity decreased. When the concentration of parasin I in CPENE and LPN increased from 1.5 to 3 mg/mL, the LDH activity decreased. However, when the concentration increased from 3 to 6 mg/mL, the LDH activity increased. These results indicated that LPNE and CPENE had no dose–dependent effect on the LDH activity. The Pickering emulsions showed lower LDH activity than parasin I, which indicated that Pickering emulsions could decrease cell damage, membrane permeability change, and cell toxicity compared with parasin I. This may be because the carrier of the Pickering emulsion and the extra compounds of chitosan or lecithin enhanced the anti-inflammatory activity and further relieved the cell damage.

**Figure 4:**
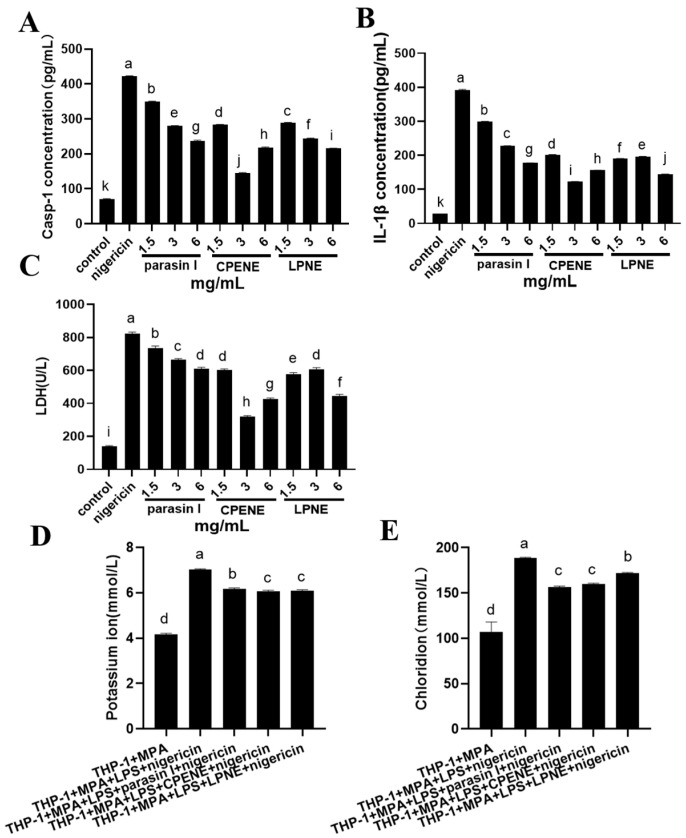
The Pickering emulsion enhances the inhibition effect of Parasin I on the activated NLRP3 inflammasome in THP-1. The casp-1 (**A**), IL-1β (**B**), LDH (**C**) and potassium (**D**) and chloride (**E**) concentration in THP-1. Different letters (“a”–“k”) indicate significant differences (*p* < 0.05).

The RAW264.7 cells are mouse peritoneal macrophages used to evaluate the inhibition of inflammation in human cells. Three kinds of Pickering emulsion were tested to show whether there is inhibition in the human acute monocytic leukemia cell line (THP-1). In Figure 4A,B, when the concentration increased from 1.5 to 6 mg/mL, parasin I showed a dose–dependent effect on the release of casp-1 and IL-1β, while LPNE had a dose–dependent effect on the release of casp-1. As for parasin I, with the increase in parasin I concentration, the casp-1 in parasin I and LPNE groups showed a lower concentration in the cell supernatant. However, the casp-1 concentration increased in CPENE when the concentration of parasin I increased from 3 to 6 mg/mL. This may be due to the disability of CPENE. When the nanoparticles’ concentration increased, the oil’s stability in water became poor and caused the nanoparticles to precipitate. The CPENE then showed a poor anti-inflammatory ability. The CPENE Pickering emulsion showed a higher inflammasome inhibition effect on LPS and nigericin in RAW264.7 or THP-1, which suggested that the Pickering emulsion had higher inhibition activity on the NLRP3 inflammasome. Udayana Ranatunga et al. [34] showed that interfacial tension in the oil-water interface could affect the interactions of nanoparticles with other components. Therefore, the nanoparticles distributed on the oil-water interface can avoid direct contact between nanoparticles and cells. Moreover, the chitosan nanoparticles may increase the anti-inflammatory activity by binding to inflammasome components.

After the anti-inflammatory activity was evaluated, the mechanism of inflammasome inhibition in Pickering and parasin I needed to be explored. The activation of the inflammasome needed the stimulus of a cascading signal. The release of potassium is generally recognized to be able to induce the activation of the inflammasome [35,36]. The chloride efflux is the downstream pathway of potassium efflux, which can cause the interaction between NEK7 and NLRP3 and activate inflammasomes (Scheme 1) [35]. After inflammasome induction, the inhibitory mechanisms of inflammasomes were explored by analyzing potassium and chloride ion release of THP-1. As shown in Figure 4E, LPS and nigericin can significantly promote IL-1β release compared with THP-1 cell and THP-1 activated by phorbol-12-myristate-13-acetate (PMA). The parasin I, CPENE and PCNE can decrease the release of potassium and chloride ions and further decrease the assembly of the NLRP3 and the secretion of IL-1β, which indicates that the CPENE and PCNE can enhance the anti-inflammatory activity by decreasing the releases of potassium and chloride ion. There was a high content of 3-glutathione- chitosan in CPENE and high content of chitosan in PCNE. This result is consistent with molecular docking results, which showed a higher bonding force in 3-glutathione-chitosan and casp-1, ALK and NLRP3. Therefore, the Pickering emulsion may also preserve the membrane potential and osmotic pressure of the macrophage and stabilize the concentration of potassium and chloride.

### 2.3. Immunofluorescence Analysis in Visceral Peritoneum

**Figure 5:**
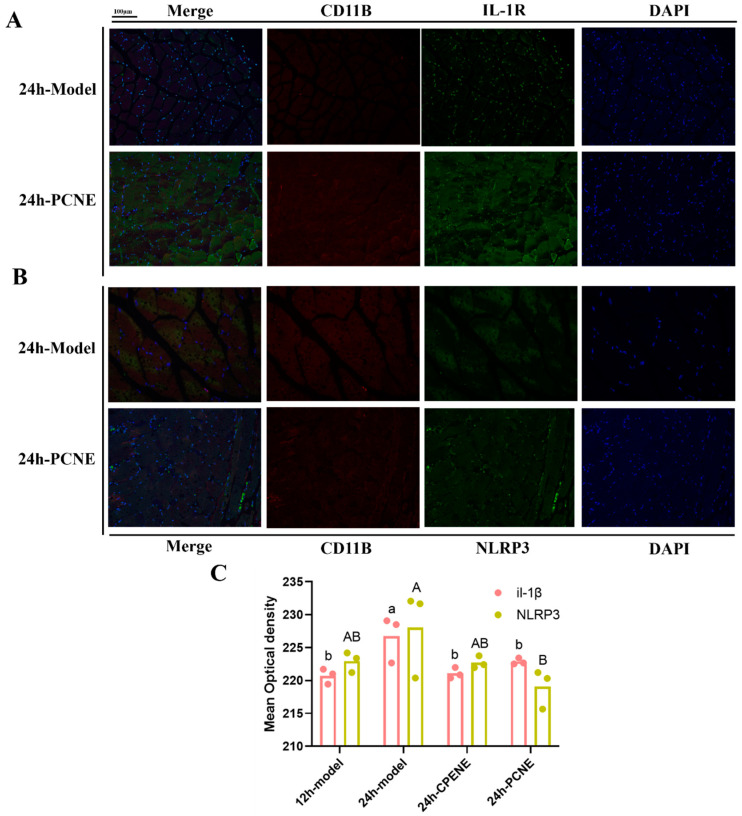
Il-1β (**A**), NLRP3 (**B**) expression and their mean optical densities (**C**) in different groups. Different letters (“a”,”b” or “A”,”B”) indicate significant differences (*p* < 0.05).

**Figure 6:**
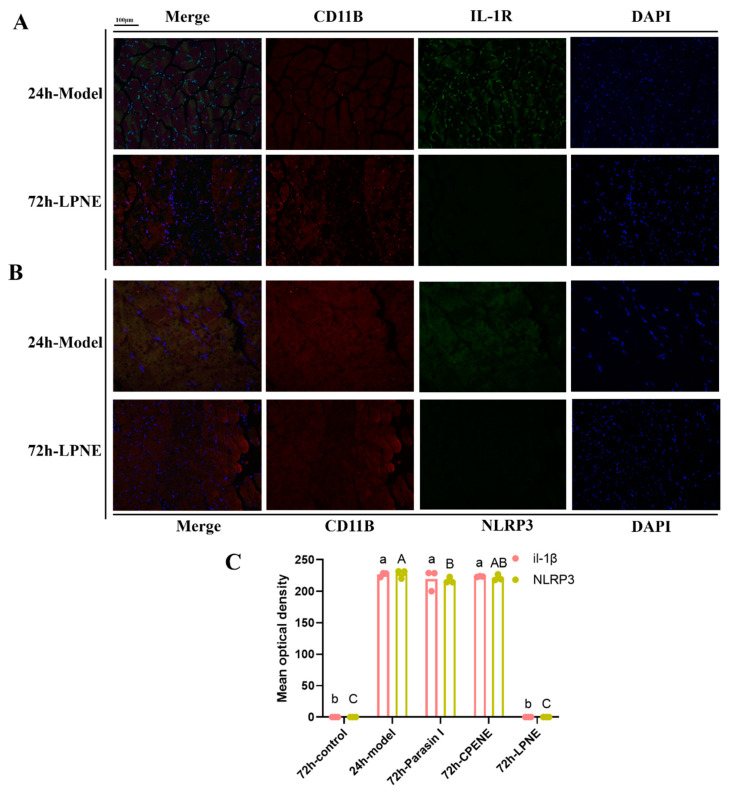
Il-1β (**A**), NLRP3 (**B**) expression and their mean optical densities (**C**) in different groups. Different letters (“a”, “b” or “A”–”C”) indicate significant differences (*p* < 0.05).

The survival of the different groups is shown in Table 2, the survival of the control group mice was 100% compared with 43.75% for control mice after intraperitoneal injection 72 h. The survival ratio of parasin I and CPENE were both 93.75%. The mechanism of Pickering emulsion improving the symptoms of inflammation was investigated by monitoring NRLP3 and IL-1β expression. In order to visualize the expression of NLRP3 and IL-1β, an immunofluorescence assay was carried out. Similar to the response to the stimulation of the *E. coli*, the peritoneum increased the expression of IL-1β and NRLP3, which were tracked with fluorescent-labeled antibodies. There was lower intensity of fluorescently labeled antibodies in the control group. The morphology of the peritoneum showed NRLP3 expression in mononuclear cells infiltrating the peritoneal membrane of Kunming mice with peritonitis. The IL-1β receptor (IL-1R1) was located on CD11B^+^ peritoneum mononuclear inflammatory cells. As shown in Figure 5, at 24 h, there were fewer CD11B^+^ peritoneum mononuclear inflammatory cells. However, IL-1β had a higher expression level in the model than that of the PCNE groups. NLRP3 also had a higher expression in the model group at 24 h than at 12 h. At 72 h, there were fewer peritoneum mononuclear inflammatory cells (Figure 6). NLRP3 and IL-1β both had higher expression except for the control and PCNE groups. NLRP3 had a relatively higher expression in the model group than parasin I and CPENE groups. These results indicated that PCNE and LPNE decreased inflammation during the treatment of peritonitis from 24 h to 72 h. They can improve the function of the visceral peritoneum by decreasing the IL-1β and NLRP3 expression.

## 3. Material and Methods

### 3.1. Reagents

The Histone H3, ECL, FITC-TSA, CY3-TSA, DAPI, and blueback fluid were purchased from servicebio Co., Ltd. (Wuhan, China). The HRP-labeled goat anti-mouse secondary antibody and HRP-labeled goat anti-rabbit secondary antibody were purchased from Wuhan Boster Biological Engineering Co., Ltd., (Wuhan, China), Fetal Bovine Serum, DMEM and penicillin-Streptomycin were purchased from GIBCO Co., Ltd., (Grand Island, NE, USA). All other chemicals used were of analytical grade.

### 3.2. Pickering Emulsion Preparation and Quantification

The methods of thiolated chitosan preparation and quantification, peptide-embedded chitosan matrices preparation, peptide-conjugated chitosan matrices preparation, chitosan peptide-embedded nanoparticles (CPEN), parasin I-conjugated chitosan nanoparticles (PCN) and lipid/peptide adduct and lipid/peptide nanoparticles (LPN) preparation, and Pickering emulsions preparation were described in detail in our previous study [22].

### 3.3. Molecular Docking

Discovery Studio 2017 was used to perform molecular docking. The 3D structure of never in mitosis gene A-related kinase 7 (NEK7), casp-1, anaplastic lymphoma kinase (ALK), NLRP3, L-α-lecithin, and chitosan were downloaded from PubChem (https://pubchem.ncbi.nlm.nih.gov/ (accessed on 1 March 2021)). The 3D structures of chitosan-GSH and chitosan-parasin I were drawn with Chemical Bio Draw Ultra 14.0 (ChemBioOffice Ultra 14.0 suite, PerkinElmer Inc., Akron, OH, USA). The parasin I, chitosan, chitosan-parasin I, or the chitosan-GSH were considered docking sites to dock with NEK7, ALK, Casp-1, or NLRP3 Dock Ligands (CDOCKER), and the lowest energy and the docking sites in the protein were calculated. The parameters in the molecular docking were set as the default values.

### 3.4. Inflammasome Suppression in Macrophages

#### 3.4.1. Inflammatory Cell Induction

To stimulate the NLRP3 inflammasome, after the frozen mouse mononuclear macrophage leukemia (RAW 264.7) cells or the human acute monocytic leukemia cell line (THP-1) were resuscitated and passaged, the cells in the logarithmic growth phase were inoculated in cell culture six-well plates and cultured overnight in a 37 °C, 5% CO_2_ incubator. The cell culture medium of RAW 264.7 and monocyte-derived macrophage THP-1 were DMEM + 10%FBS + 1% (Penicillin-Streptomycin Solution) and RPMI 1640 + 10%FBS + 1% (Penicillin-Streptomycin Solution), respectively. The cells were incubated with LPS (50 μg/mL) for 3 h. Subsequently, the cells were incubated with CPENE, PCNE, or parasin I for 0.5 h, with nigericin (10 μM) for 0.5 h. The blank group was regarded as the control group (RAW 264.7 or THP-1 treated with LPS (50 μg/mL) for 3 h and nigericin (10 μM) for 0.5 h). The cell supernatant was obtained after centrifuging for 10 min at 3000 rpm. The cell supernatant was prepared for the following measurements. The activity of lactate dehydrogenase (LDH) was analyzed by a LDH assay kit using cell supernatant.

#### 3.4.2. Chloride Ion Concentration Detection

The chloride ion in the supernatant of RAW 264.7 and THP-1 was measured by a chloride ion assay kit (Nanjing Jiancheng Bioengineering Institute (Nanjing, China), C003-2). A volume of 10 μL deionized water, chloride standard solution, or cell supernatant was added to the 250 μL mercury thiocyanate working solution. After 5 min, the OD value was recorded at 480 nm using a microplate. The standard curve calculated the chloride ion concentration of the supernatant.

#### 3.4.3. Potassium Ion Concentration Detection

The potassium ion in the supernatant of RAW 264.7 and THP-1 was measured by a potassium ion assay kit (Nanjing Jiancheng Bioengineering Institute (Nanjing, China), C001-2). A volume of 50 μL deionized water, standard potassium solution, or cell supernatant was added to the 200 μL NA-TPB working solution. After 5 min, the OD value was recorded at 450 nm using a microplate. The standard curve calculated the potassium ion concentration of the supernatant.

#### 3.4.4. Enzyme-Linked Immunosorbent Assay (Elisa)

The IL-1β and casp-1 concentration in the supernatants of RAW 264.7 and THP-1 were assayed by human IL-1β (Elabscience Biotechnology Co., Ltd., E-EL-H0149c, Wuhan, China) and casp-1 (Elabscience Biotechnology Co., Ltd., E-EL-H0016c, Wuhan, China) Elisa kit or mice IL-1β (Elabscience Biotechnology Co., Ltd., E-EL-M0037c, Wuhan, China) and casp-1 (Elabscience Biotechnology Co., Ltd., E-EL-M0201c, Wuhan, China) Elisa kit according to the manufacturer’s instructions.

#### 3.4.5. Therapy of Mice Peritonitis Model

The therapy and peritonitis modelling was carried out according to the methods of our previous studies. The 112 Kunming mice (7 weeks old, 7 groups each containing 8 male and 8 female animals, 18–22 g) were bought from the laboratory animal center of China Three Gorges University. After being bred under 12 h light/dark cycles with access to standard meals and water, the mice were intraperitoneally injected with *E. coli* ATCC 25922 (1 × 10^5^ CFU/mL, 10 mL/kg) except for the blank control group. The dosage of *E. coli* was chosen following the preliminary experiment. After 1 h, the parasin I solution, ciprofloxacin (CPFX) solution, and three Pickering emulsions (100 mg/mL) were injected intraperitoneally. Mice injected with saline served as negative control groups. The survival of mice was recorded after 6, 12, 24, and 72 h. The experiments were conducted following the National Research Council Guide for the Care and Use of Laboratory Animals and approved by the Hubei Academy of preventive medicine Ethics Committee. The assigned accreditation number of the laboratory was 20191825.

#### 3.4.6. Tissue Staining and Immunohistochemistry

Tissue samples were firstly fixed in 4% paraformaldehyde phosphate tissue fixative. After fixation, the samples were washed with phosphate buffer solution and then immersed in a series of ethanol solutions with increasing concentration for dehydration. The samples were then further dehydrated overnight using an automatic tissue dehydrator. Subsequently, the tissue samples were embedded in paraffin on the paraffin embedding machine and properly cooled on the cold table. The paraffin section of the peritoneum was dewaxed and washed with water, and the antigen was repaired with EDTA antigen repair buffer (pH 8.0) in a microwave oven. The section was circulated with histochemical strokes and sealed with hydrogen peroxide and BSA. The primary antibodies (CD11B + NLRP3 or CD11B + IL-1R, Servicebio, bs-20697r) and the HRP-labeled secondary antibodies (HRP RAB CY3TSA 488 goat anti-rabbit, Servicebio, Gb25303, Wuhan, China) were added to the section in sequence, and incubated 30 min and overnight, respectively. After incubation, the antigen was heated in EDTA antigen repair buffer (pH 8.0) in the microwave to remove the primary and secondary antibodies. Then the second primary and secondary antibodies were added to the slices, and DAPI to stain the nucleus. After self-fluorescence quenching and sealing with anti-fluorescence tablets, the section was analyzed using a fluorescence microscope.

## 4. Conclusions

The food-derived Pickering emulsions stabilized by antimicrobial peptide nanoparticles were tested as therapeutic agents for bacterial infectious diseases in silico, in vitro and in vivo. CPENE significantly improved the NLRP3 activation and maturation of the IL-1β in RAW 264.7 and THP-1 cells. LPNE showed a dose–dependent inhibition effect on the release of IL-1β and casp-1. When the concentration of parasin I increased from 1.5 mg/mL to 3 mg/mL, the LDH activity decreased in the CPENE and LPNE groups. However, from 1.5 to 6 mg/mL, LPNE had a dose–dependent effect on the release of casp-1. CPENE and PCNE can decrease the release of the potassium and chloride ion. At 24 h, there were fewer CD11B^+^ peritoneum mononuclear inflammatory cells in all peritoneum groups. Therefore, LPNE may inhibit the activation of the inflammasome by decreasing LDH activity and potassium and chloride ions through binding with components of NLRP3.

## Figures and Tables

**Table 1:** The interaction force and energy between NLRP3 components with chitosan, lecithin, or parasin I.

Name	CDOCKER_INTERACTION_ENERGY (kcal/mol)
NEK7-L-α-Lecithin	−79.3303
NEK7-Chitosan	−93.7484
Casp-1-Chitosan-glutathione-3	−97.0533
Casp-1-L-α-Lecithin	−70.3612
ALK-Chitosan-glutathione-3	−106.754
ALK-L-α-Lecithin	−79.8664
NLRP3-3-glutathione-chitosan-parasin	−167.167
NLRP3-parasin I	−138.395

**Table 2:** The survival of mice in different groups at 0, 12, 24, 72 h.

Time (h)	0	12	24	72
Control	16	16	16	16
Model	16	13	11	7
CPFX	16	16	16	16
Parasin I	16	16	16	15
CPENE	16	16	15	15
LPNE	16	15	14	13
PCNE	16	14	14	12

## Data Availability

Not applicable.
